# Short Term Effect of Calcium Hydroxide, Mineral Trioxide Aggregate and Calcium-Enriched Mixture Cement on the Strength of Bovine Root Dentin

**Published:** 2012-06-01

**Authors:** Safoora Sahebi, Mohammadreza Nabavizadeh, Vahid Dolatkhah, Davoud Jamshidi

**Affiliations:** 1. Department of Endodontics, Dental school, Shiraz University of Medical Sciences, Shiraz, Iran; 2. Endodontist, Shiraz, Iran; 3. Postgraduate Student of Endodontics, Dental school, Shiraz University of Medical Sciences, Shiraz, Iran

**Keywords:** Calcium Hydroxide, Calcium-Enriched Mixture, Cem Cement, Dentin, Mineral Trioxide Aggregate

## Abstract

**Introduction:**

Some studies in dental literature have proposed that short term and long term exposure of root dentin to calcium hydroxide predisposes it to fracture. Mineral trioxide aggregate (MTA) and a recently introduced endodontic material, calcium-enriched mixture (CEM) cement may be used instead of calcium hydroxide and might have an effect on the strength of root dentin. Therefore, the aim of this in vitro study was to compare the short-term effect of calcium hydroxide, MTA and CEM cement on the strength of bovine root dentin.

**Materials and Methods:**

In this experimental study, 15 freshly extracted intact bovine incisors were selected. A cylinder with uniform wall thickness (internal diameter of 2.5 and external diameter of 5.5) was prepared. The cylinders were cut longitudinally into 4 symmetrical pieces. The 60 prepared samples were divided into four groups (n =15). The samples were placed in 4 petri-dishes containing calcium hydroxide, MTA, CEM cement and normal saline as the control group. They were then subjected to flexural forces applied by Instron universal machine. Data was analyzed using ANOVA and Tukey test.

**Results:**

The mean flexural force in the calcium hydroxide, MTA and CEM cement groups was significantly lower than that in the control teeth (77.9 N, 90.66 N, 94.40 N, compared to 125.12 N respectively, P=0.001). There were no significant differences between calcium hydroxide, MTA and CEM cement group.

**Conclusion:**

MTA and CEM cement decreased the flexural strength of bovine root dentin, like their counterpart calcium hydroxide. Further studies are required to determine the effect of these materials on human root dentin clinically.

## Introduction

Dentin is an energy-absorbing cushion for enamel and an effective protecting barrier for the pulp [1]. It is made of hydroxyapatite and collagen. Because of its composite nature and its structural design, it is much tougher than hydroxyapatite/collagen [2].

Historically many endodontic materials had been used in root canal system as an intracanal medicament that may have affected dentin strength. Calcium hydroxide (CH) has been used for disinfection of root canals as well as apexogenesis and apexification procedures for a long period [3]. It has also been used as a liner underneath restorations, as a pulp capping material [4][5] and for control of inflammatory root resorption [6]. It is the most popular inter-appointment medicament [7]. CH has the ability to dissolve organic tissues [8] and to kill bacteria [9][10]. Its broad spectrum antimicrobial activity is one of the reasons why it is popular [11][12]. CH creates an alkaline environment inside dentinal tubules and as a result shows antibacterial properties and stimulates hard tissue barrier formation [13].

There are a few reports in literature about the changes in the strength of root dentin in response to endodontic materials. White et al. reported a reduction in the strength of bovine root dentin approximately 32%, 33% and 59% after exposure to CH, mineral trioxide aggregate (MTA) and hypochlorite, respectively [14]. It was shown that CH can disturb the hydroxyapatite and collagen network of dentine and as a consequence be susceptible to fracture [15]. The strength of dentin was reduced significantly following 1 month exposure to CH [16]; CH can reduce the microtensile fracture strength of dentin as much as 23-43.9% [17].

MTA was first introduced in 1993 by Torabinejad et al. as a root end filling material and for repair of lateral root perforations [18]. Since then it has been used as apical barrier, perforation repair in many parts of tooth, as a pulp cap and pulpotomy agent, and for treatment of root resorption and as a root filling material [19][20]. Similar to CH it has high alkaline pH when freshly mixed [21]. There are few studies that look at the effect of MTA on the strength of root dentin. White et al. reported 33% decrease in the strength of bovine root dentin after 5 week exposure to MTA [14]. The immature sheep teeth that were filled with MTA were significantly stronger than those which had been filled with CH [22].

Recently, calcium-enriched mixture (CEM) cement has been introduced with the same clinical application as MTA but with a different chemical composition [23]. It exhibits high alkaline pH and release CH [24][25]; CEM also has good antimicrobial properties [26].

A study proposed that the proteolytic action of CH could weaken a tooth and predispose it to fracture [27]. Therefore, it could also be possible for other CH releasing agents such as MTA or CEM cement to weaken root dentin. Numerous studies have shown that CH can reduce the strength and hardness of root dentin. There are a few reports in literature about the effect of MTA on the strength of root dentin, these have contradictory results. There are no studies which analyze the effect of CEM cement on the strength of root dentin. Therefore, this in vitro study was designed to compare the effect of these materials on the strength of root dentin.

## Materials and Methods

An in vitro model for preparation of dentin test specimens, originally described by Haapasalo and Ørstavik [28] with some modifications, was used. Freshly extracted intact bovine incisors were selected for the study. The teeth were stored in physiologic saline until use, to prevent dehydration. In order to maintain a 15-mm portion of the roots, the apical 5-mm and two third of the crowns of the teeth were separated with a water cooled, rotating diamond saw (D & Z, Wiesbaden, Germany) at 1000 rpm. The end of the roots was embedded in acrylic resin in order to do the process of preparing the desired cylinder. A 2.5 mm diameter twist drill was used to eliminate the canal and a cylinder of uniform wall thickness was made with a water cooled bone biopsy hole saw at low speed. External diameter of 5.5 mm was made with a 3 mm cylindrical carbide bur (D & Z, Wiesbaden, Germany).

**Figure 1:**
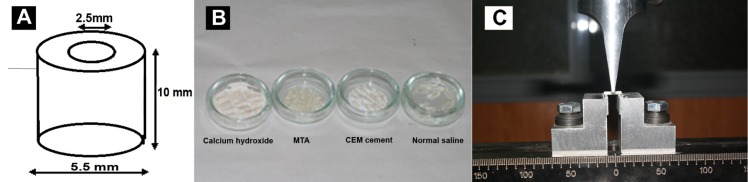
A) Appearance and dimensions of a prepared dentin cylinder. B) Four petri-dishes containing the calcium hydroxide, MTA, CEM cement and control group. C) A dentin sample under the load by IUM

To verify the diameters of the cylinder a digital caliper (Mitutoyo, Kawasaki, Kanakawa, Japan) was used. Ultimately, this procedure produced a symmetrical cylinder of dentin of 5.5-mm outer diameter, 2.5-mm inner diameter, and 10-mm long (Figure 1A).These cylinders were cut longitudinally into four symmetrical pieces with the use of a rotating thin diamond saw. Then, the 10-mm long specimens were separated from acrylic resin with the diamond saw. Also all the samples were then weighed on a Mettler balance (Mettler Instrument Company, Highstown, NJ, USA) to verify accuracy of slices. All of the 60 samples were randomly divided into 4 groups with 15 specimens each. The dentin samples were then preserved in normal saline to prevent dehydration. Calcium hydroxide (Merck, Darmstadt, Germany), MTA (Dentsply, Tulsa, OK, USA) and CEM cement (BioniqueDent, Tehran, Iran) were prepared based on the manufacturer instructions. The canal surface of dentin bars were then placed into one of four Petri dishes containing a 2-mm depth of calcium hydroxide, MTA, CEM cement or physiologic saline (Figure 1B).

The samples were kept in the dishes for 30 days; normal saline was added to dishes as needed to keep the moisture. At the end of this period, each sample was rinsed with saline. Sixty dentin specimens were subjected to three-point bending test, using a universal testing machine (Z010, Zwick, Ulm, Germany) with blade size of 1 mm (Figure 1C). For the testing apparatus a sample holder with two rectangular supports was used.

**Figure 2:**
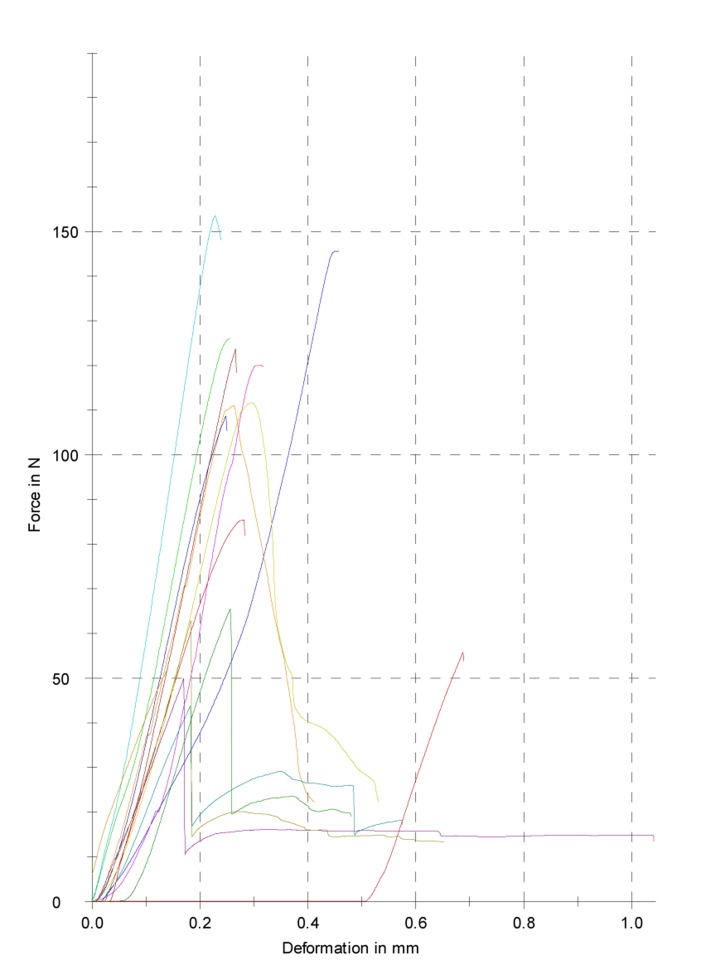
Diagram illustrating the amount of force required to fracture the dentin samples in CEM group. Each line represents CEM specimen.(n=15)

Samples were placed with the canal surface centered on the support. The load testing machine was run at a cross-head speed of 1 mm min^-1^, the forces were applied in a vertical direction and the samples were tested until fractured. The zwich/roell software was used to record data on a plotter to give load displacement curves (Figure 2). Maximum force at the point of fracture was recorded. For comparison of the groups in terms of maximum force at the point of fracture, a one way ANOVA and Tukey tests were used. Statistical calculations were performed with the SPSS software. In order to assess the assumption of normality Kruskal-Wallis test was used for each group separately.

## Results

There was no significant departure from normality assumption (P>0.05). Because the group samples were equal, Hartley test was applied to assess the homogeneity of variances of different groups. There was no significant evidence of heterogenic (P>0.05).

The mean maximum forces required to fracture the samples in the CH, MTA, and CEM is presented in Table 1. CH, MTA and CEM cement decreased the strength of dentin by 38, 28 and 25%, respectively. There was no statistically significant difference among the 3 groups (P=0.45); however, there was significant difference between experimental and control groups (P<0.05).

**Table 1:** Maximum force means (SD) of the four groups required to cause dentin fracture

**Group**	**Mean (SD)**	**Max**	**Min**
**Ca(OH)_2_**	77.99 (25.56)	122	37.80
**MTA**	90.66 (26.87)	149	54.20
**CEM**	94.40 (37.01)	153	43.70
**Normal Saline**	125.12 (29.86)	191	91.8

## Discussion

Bovine teeth were selected for this study. They are readily available and share basic microscope morphologic qualities [14]. They are a good substitute for human dentin. Schilke et al. did not observe any significant differences in dentinal tubule diameters in human and bovine dentin by scanning electronic microscopy (SEM).

Tubular density found in bovine root was 23738-4457 tubules/mm^2^ in human dentin [29].

The strength of root dentin has been evaluated by shear flexural and compressive tests [14]. Researchers have used shear forces [14][17][22][27], compressive forces [16][30] and flexural test [31][32] to examine the dentin samples. In this study the flexural forces applied by the cross-head of the Instron universal machine on the dentin samples was assessed.

Short-term use of CH, MTA and CEM reduced the strength of bovine root dentin by approximately 38%, 28% and 25% after 30 days, respectively. This reduction of the strength of root dentin by CH is consistent with the report by Rosenberg et al. [17] on human dentin and other reports of animal studies [14][27][33]. It has been proposed that CH, because of its alkaline pH, can cause breakdown of protein structure of dentin and therefore weaken the structure of dentin [27]. The weakening of dentin by MTA and CEM cement might be to the gradual release of CH. Since the concentration of released CH from MTA might be lower than that of CH itself, the breakdown of protein structure by it might be lower than that of CH. It has been reported that MTA produces a mechanical and chemical bonding with the dentin wall [34]. This phenomenon might compensate for the 28% decrease in the strength of dentin observed in our study and therefore the clinical significance of this 28% decrease by MTA might be lower than that of the CH.

There have been numerous studies showing the weakening of the structure of root dentin by CH. The result of this study is consistent with the report of Andreasen et al. [27] who demonstrated the weakening of tooth root dentin. In a 1 week exposure to saturated CH, Grigoratos et al. [35] reported reduction of the flexural strength of standardized dentin bars. Rosenberg et al. [17], in measuring the effect of CH root filling on the micro-tensile fracture strength of teeth, reported weakening of root structure by as much as 43.9%. This study confirmed the work of White et al. [14] who reported the reduction of the strength of bovine root dentin after exposure to CH and MTA as much as 32% and 33%, respectively. Our study is consistent with another study. [15] which demonstrated that exposure of bovine dentin to CH can make it prone to fracture. However, it is inconsistent with the work of Doyon et al. [30] who showed 30 day exposure to CH had no significant effect on the fracture resistance of dentin disks. Hatibovic-Kofman et al. [22] showed the fracture strength of roots filled with CH decreased over time. In 2010, others [16] concluded that 30 days application of CH reduced the compressive strength of human root dentin by almost 15% after 30 days. In summary, the result of our study confirmed the result of previous studies about weakening action of CH on the strength of root dentin [17][27][30].

There are few studies that present conflicting results regarding the effect of MTA on the strength of root dentin. White et al. [14] showed a 33% reduction of bovine root dentin after exposure to MTA which is consistent with our results. Hatibovic-Kofman et al. [22] showed that fracture resistance of sheep teeth that were treated with MTA decreased 20% at 30 days which is in consistent with present study results. Although they found the fracture resistant increased 18% after 12 months and the total decrease is only 2% in one year. We propose that there should be more studies with different and extended exposure times to determine the effect of MTA on the strength of root dentin.

CEM cement exhibits high alkaline pH and after setting it can release CH [25]. Since CEM is a novel biomaterial there studies have not yet determined the effect of this endodontic biomaterial on the strength of root dentin. Our study is the first that showed CEM cement can decrease the strength of bovine root dentin by almost 25%. Other studies that looked at the properties of CEM compared to MTA have showed that CEM has less setting time, more flow and less film thickness compared with MTA [36], with the ability to produce hydroxyapatite on its surface [24]. Unlike CH, CEM cement is not toxic [37]. Animal studies have shown the regeneration of periodontal ligament and cementum when adjacent to CEM cement [38][39]. It has antimicrobial properties like CH and superior to MTA [26]. There was no difference between the effect of MTA and CEM on the strength of root dentin. This might be due to the similar pattern of releasing CH by these biomaterials.

Teeth were extracted from the jaws of the cows and immediately stored in a physiologic environment; throughout the study we tried to keep the dentin samples in a physiologic environment to eliminate compounding factors which might influence the structure of dentin samples. However, in previous studies dentin samples had been stored in chloramine-T solution and formalin [17][22][27]. These solutions might influence the root dentin and cause different results.

## Conclusion

Under the limitation of this in vitro we can conclude that CH, MTA and CEM cement decrease the flexural strength of bovine root dentin. Further studies are required to determine the effect of these materials on human root dentin in the real clinical situation.
